# Oxidative Degradation of Tetracycline by Magnetite and Persulfate: Performance, Water Matrix Effect, and Reaction Mechanism

**DOI:** 10.3390/nano11092292

**Published:** 2021-09-03

**Authors:** Deokhui Lee, Soyeon Kim, Kai Tang, Michael De Volder, Yuhoon Hwang

**Affiliations:** 1 Department of Environmental Engineering, Seoul National University of Science and Technology, Seoul 01811, Korea; siksik9999@seoultech.ac.kr (D.L.); sopia1290@seoultech.ac.kr (S.K.); 2 Department of Environmental Engineering, Technical University of Denmark, 2800 Konges-Lyngby, Denmark; kait@env.dtu.dk; 3 Institute for Manufacturing, Department of Engineering, University of Cambridge, Cambridge CB3 0FS, UK; mfld2@cam.ac.uk

**Keywords:** magnetite, sulfate radicals, tetracycline, heterogeneous fenton reaction, micropollutants

## Abstract

This study presents a strategy to remove tetracycline by using magnetite-activated persulfate. Magnetite (Fe_3_O_4_) was synthesized at high purity levels—as established via X-ray diffractometry, transmission electron microscopy, and N_2_ sorption analyses—and tetracycline was degraded within 60 min in the presence of both magnetite and persulfate (K_2_S_2_O_8_), while the use of either substance yielded limited degradation efficiency. The effects of magnetite and persulfate dosage, the initial concentration of tetracycline, and the initial pH on the oxidative degradation of tetracycline were interrogated. The results demonstrate that the efficiency of tetracycline removal increased in line with magnetite and persulfate dosage. However, the reaction rate increased only when increasing the magnetite dosage, not the persulfate dosage. This finding indicates that magnetite serves as a catalyst in converting persulfate species into sulfate radicals. Acidic conditions were favorable for tetracycline degradation. Moreover, the effects of using a water matrix were investigated by using wastewater treatment plant effluent. Comparably lower removal efficiencies were obtained in the effluent than in ultrapure water, most likely due to competitive reactions among the organic and inorganic species in the effluent. Increased concentrations of persulfate also enhanced removal efficiency in the effluent. The tetracycline degradation pathway through the magnetite/persulfate system was identified by using a liquid chromatograph-tandem mass spectrometer. Overall, this study demonstrates that heterogeneous Fenton reactions when using a mixture of magnetite and persulfate have a high potential to control micropollutants in wastewater.

## 1. Introduction

Pharmaceuticals such as antibiotics, which are highly water-soluble and hardly biodegradable, have been widely detected in aquatic systems in recent decades, resulting in serious threats to both public health and the natural environment, due not only to their toxicity but also to the prevalence of bacteria that have become antibiotic-resistant as a result of their heavy use [1,2]. Among a number of antibiotics, tetracycline (TC) is one of the most widely used in veterinary medicine and causes many of the environmental problems described above [3,4].

Conventional biological wastewater treatment processes, such as activated sludge, are relatively inefficient at removing TC [5,6]. According to Oulton et al. (2010), the reported removal efficiency of TC by activated sludge varies widely, ranging from 23% to 91%. Advanced oxidation processes (AOPs) have recently been proven efficient for removing non-biodegradable organic substances, including TC [7]. The primary mechanism behind AOPs involves generating highly reactive free radicals and then exploiting these species to degrade organic compounds oxidatively [8]. The Fenton process is a well-known AOP in which hydroxyl radicals are generated via reactions between iron salts and hydrogen peroxide (Fe^2+^/H_2_O_2_) [9]. However, it has certain limitations, including the inefficient utilization of rapidly formed hydroxyl radicals and the ineffective removal of organic pollutants [10]. AOPs involving sulfate radicals (SO_4_^•^^−^) have gained great attention as a means of overcoming these limitations, since they have redox potentials of 2.5 to 3.1 V, higher than those of hydroxyl radicals (1.8 to 2.7 V). In addition, sulfate radicals live longer than hydroxyl radicals and are more selective at removing pollutants. Their operating pH is also much wider than hydroxyl radicals [11,12]. Persulfate (PS, S_2_O_8_^2−^) is stable at room temperature and can be activated by using heat, ultraviolet (UV), and transition metals (e.g., Fe^2+^ and Co^2+^) to form highly reactive sulfate radicals, as described in Equation (1) below [13]:

S_2_O_8_^2−^ + activator → SO_4_^•−^+ (SO_4_^•−^ or SO_4_^2−^) (1)

Specifically, the reaction mechanism of PS activated by Fe^2+^, as explained in Equations (2) and (3) [14], is similar to the Fenton process, because the molecular structure of PS is an asymmetrically substituted derivative of hydrogen peroxide [15]:

S_2_O_8_^2−^ + Fe^2+^ → SO_4_^•−^ + Fe^3+^ + SO_4_^2−^ (2)

SO_4_^•−^ + Fe^2+^ → Fe^3+^ + SO_4_^2−^ (3)

Although the Fe^2+^/PS process has been improved in terms of redox potentials and applicable operating pH when compared to the classic Fenton process, it still has several inherent drawbacks that have limited its broad application in wastewater treatment. First, excess Fe^2+^ causes SO_4_^•−^ scavenging, which in turn suppresses oxidation of the target contaminant via the mechanism described in Equation (3). Second, Fe^2+^ activation only occurs effectively under acidic pH conditions (2–4), in order to prevent rapid iron precipitation such as the formation of ferric hydroxy complexes when the pH is above 4. Moreover, at the end of the Fe^2+^/PS process, a large amount of iron sludge is yielded, thereby leading to additional cost in treating it before discharge [16,17]. 

Heterogeneous Fenton systems that use iron-containing solids (Fe_2_O_3_, Fe_3_O_4_, FeO(OH), etc.) as a source of ferrous iron have been introduced to overcome the limitations of homogeneous Fenton processes [18]. With the use of a heterogeneous Fenton process, the rate of sludge production can be regulated, and the operating pH can be wider than in the homogeneous Fenton process. Magnetite (Fe_3_O_4_) nanoparticles are considered effective catalysts for heterogeneous Fenton processes, because Fe^2+^ of Fe_3_O_4_ has octahedral sites that support the decomposition of H_2_O_2_ or PS into reactive radicals [19,20]. In addition, Fe_3_O_4_ nanoparticles can be easily manufactured, have high biocompatibilities and stabilities, and are easily separated by using an external magnetic field. These attributes make them highly suitable as catalysts in the removal of non-degradable organic substances [21,22]. 

Table 1 summarizes the experimental conditions used in this research compared with other studies reported in the literature. Heterogeneous Fenton processes that employ Fe_3_O_4_ and PS have been reported to degrade several pharmaceutical compounds, such as norfloxacin and sulfamethoxazole [23,24]. The use of nanocomposite materials in which the magnetite was combined with other supporting materials, such as biochar, activated carbon, and chitosan for degradation of tetracycline, was also reported [25,26,27,28]. Several previous works have reported the inhibitory effect of organic and inorganic substances individually [21,25,26], but there is no report on the inhibitory effect of actual wastewater samples on TC degradation by the Fe_3_O_4_/PS system. Thus, the practicality of a heterogeneous Fenton process that uses Fe_3_O_4_ and PS in real-world applications has yet to be tested. 

This study evaluates the capacity of Fe_3_O_4_ as a catalyst for activating PS to the extent that it can degrade TC. Fe_3_O_4_ nanoparticles were synthesized and subsequently characterized by using nitrogen sorption via Brunauer–Emmett–Teller (BET) analyses, X-ray diffraction (XRD), and transmission electron microscopy (TEM). The removal efficiency of TC under various reaction conditions was investigated. Reaction variables include Fe_3_O_4_ and PS concentration as well as the initial pH. The effect of the water matrix was evaluated by comparing removal efficiencies from deionized water and wastewater treatment plant effluent. The TC degradation mechanism was further investigated by analyzing the reaction intermediate, using the liquid chromatograph-tandem mass spectrometer.

## 2. Materials and Methods

### 2.1. Chemicals

Reagent-grade FeCl_2_·4H_2_O (99%), FeCl_3_·6H_2_O (97%), NH_4_OH (25~30%), K_2_S_2_O_8_ (98%), HClO_4_ (≥70%), ethanol [C_2_H_5_OH] (99%), and H_3_PO_4_ (99%) were purchased from Samchun Chemical (Seoul, Korea). NaOH (≥93%, reagent grade) was purchased from SHOWA KAKO Corporation (Osaka, Japan). Tetracycline hydrochloride [C_22_H_25_N_2_O_8_Cl] (≥95%, analytical grade), acetonitrile (C_2_H_3_N, HPLC grade), methanol (CH_3_OH, HPLC grade), and formic acid (CH₂O₂, HPLC grade) were purchased from Sigma-Aldrich (St. Louis, MO, USA). Ultrapure water was produced by a water purification system (Synergy^®^, Merck, Kenilworth, NJ, USA)

### 2.2. Fe_3_O_4_ Preparation

Fe_3_O_4_ was prepared by the coprecipitation method under alkaline conditions according to Equation (4) [29]

2FeCl_3_(aq) + FeCl_2_(aq) + 8NH_3_(aq) → Fe_3_O_4_(s) + NH_4_Cl(aq) (4)

First, ultrapure water was purged with N_2_ gas for 1 h. 10 mL of 3.0 M FeCl_2_·4H_2_O (in 2.0 M HCl) and 10 mL of 6.0 M FeCl_3_·6H_2_O (in 2.0 M HCl) solutions were prepared as starting materials. An aqueous solution of ferric chloride and ferrous chloride at a 2:1 molar ratio of Fe^3+^: Fe^2+^ was prepared, injected into a 1 L three-necked round flask containing 600 mL deionized water, and heated to 50 to 60 °C with stirring. After 10 min, 200 mL of 2.8 M NH_4_OH was added dropwise at a constant flow rate for 45 min, and the pH of the aqueous solution was raised to 10–11. The resulting solution was then heated and stirred at 50 to 60 °C for one hour and then stirred without heating for an additional hour. The reaction was continuously purged with N_2_ gas during all of the synthesis steps described above. The resulting black solid was separated with a neodymium magnet, washed with ultrapure water until it assumed a neutral pH, and then washed three times with methanol. Finally, the product was dried overnight at 50 °C in a vacuum oven and stored in an anaerobic chamber prior to use in experiments.

### 2.3. Fe_3_O_4_ Characterization

The crystal structure of Fe_3_O_4_ was analyzed by X-ray diffractometer (XRD, D8 ADVANCE, Bruker, Billerica, MA, USA). Morphology and size were observed using a transmission electron microscope (TEM, JEM-2010, JEOL, Tokyo, Japan). The specific surface areas of the Fe_3_O_4_ particles were obtained by applying BET analyses to N_2_ sorption data acquired at 77 K and p/p_0_ = 0.99 (BELSORP-mini II, MicrotracBEL, Osaka, Japan).

### 2.4. TC Degradation Experiment Using Fe_3_O_4_/PS

Fifty milliliters of 41.6 μM of TC dissolved in ultrapure water was transferred to a glass vial. Fe_3_O_4_ and PS were added into the glass vial simultaneously at the start of the experiment; therefore, this study did not consider the equilibrium time for TC adsorption on the catalyst. The solution was continuously mixed using a vertical rotating mixer (VM-80, Miulab, Zhejiang, Hangzhou, China) at a speed of 20 rpm. The batch experiments were conducted by adjusting the initial concentrations of Fe_3_O_4_ and PS from 0.2 to 1 g/L and 0.05 to 1 mM, respectively, to investigate the impacts of Fe_3_O_4_ and PS concentration on TC removal. To examine the effect of the initial pH on TC removal, it was adjusted by using 0.1 M NaOH or 0.1 M HClO_4_ over a range of 3 to 9. As ClO_4_^−^ has little impact on the oxidation process, it is a more appropriate ionic species for adjusting the pH than HCl and H_2_SO_4_ [23]_._ The reaction time for each batch experiment was 60 min, during which a 1-mL sample was collected from each vial at specified time intervals, immediately filtered with a 0.45 μm PES syringe filter, and then quenched by adding 0.1 mL of ethanol to prevent further side reactions prior to analysis [30]. TC concentration over the experimental time was plotted by a pseudo first-order kinetic equation:

C_t_ = C_0_·exp(−k_obs_·t) (5)

where k is the observed rate constant (min^−1^), C_0_ is the initial concentration of TC, and C_t_is the concentration of TC at time t. 

To understand the formation of intermediate product of TC during its reaction with Fe_3_O_4_/PS, a batch experiment was also conducted. TC, PS, and Fe_3_O_4_ were added into a glass beaker with 200 mL of ultrapure water, resulting in initial concentrations of 41.6 μM, 1 mM, and 1 g/L respectively. pH was adjusted to 4.7. Samples were taken at 0, 10, 20, 30, 40, 50, and 60 min, filtered with a 0.45 μm PES syringe filter, and 0.1 mL of ethanol was added into a 0.9 mL sample to quench the reaction.

To examine the effect of the water matrix on TC removal by Fe_3_O_4_/PS, batch experiments using ultrapure water or municipal effluent wastewater from a regional wastewater treatment plant (WWTP) were performed and their results compared. The common parameters of the effluent are presented in the supporting information (Table S1).

### 2.5. Analytical Methods

For the quantitation analysis in the degradation experiments, TC concentration was analyzed by high-performance liquid chromatography (HPLC, U-3000, Thermo Fisher, Waltham, MA, USA). TC was separated in chromatography by using a C18 column (Accucore AQ 4.6 × 150 mm, 2.6 μm, Thermo Fisher, Waltham, MA, USA) and was detected at a 280 nm absorbance wavelength. The oven temperature was set at 50 °C. The mixture of 0.1% phosphoric acid/acetonitrile (85:15, *v*/*v*) was used as isocratic elution at a flow rate of 1 mL/min. The sample injection volume was 10 μL, while the transformation products of TC were quantified by HPLC (Vanquish Flex, Thermo Fisher Scientific, Waltham, MA, USA) coupled with a triple quadrupole mass spectrometer (TSQ Quantis, Thermo Fisher Scientific, Waltham, MA, USA). Chromatographic separation was performed using a C18 column (4.6 × 50 mm, 3.5 µm, Agilent, Santa Clara, CA, USA). A detailed description of the method can be found in Text S1.

A spectrofluorometer (F-7000 Fluorescence Spectrophotometer, Hitachi, Tokyo, Japan) was used to investigate changes in fluorescence intensity for the samples during the heterogeneous Fenton reaction.

## 3. Results

### 3.1. Physical and Morphological Characteristics of Fe_3_O_4_

**Figure 1:**
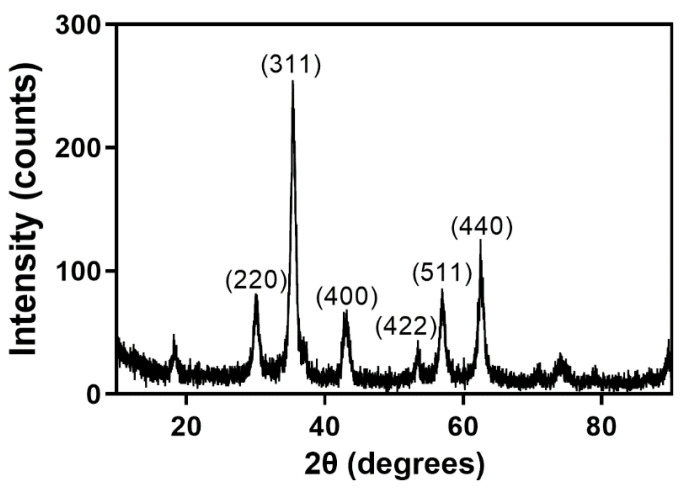
XRD patterns of Fe_3_O_4_ nanoparticles.

XRD analyses were conducted to understand the crystallinity of the synthesized Fe_3_O_4_ nanoparticles. The diffraction results, as shown in Figure 1, clearly demonstrate that the Fe_3_O_4_ materials are crystalline, thus indicating a successful synthesis. The XRD patterns of the synthesized Fe_3_O_4_ have peaks at 2θ of 30.06, 35.43, 43.04, 53.55, 57.17, and 62.73, which are assignable to (220), (311), (400), (422), (511), and (440) of cubic Fe_3_O_4_, respectively, according to the Joint Committee for Powder Diffraction Studies (JCPDS No. 19-0629) [26]. In addition, the positions of the diffraction peaks indicate an inverted spinel structure [31]. The average Fe_3_O_4_ particle size can be obtained from the XRD pattern via the Debye-Scherrer formula D = Kλ/βcos(θ), where K is the Scherrer constant (0.89), λ is the X-ray wavelength (1.54 Å), β is full width at the half maximum of a given peak, and θ is the Bragg diffraction angle [17]. Application of the Debye-Scherrer equation to the most intense peak, i.e., the (311) reflection (Figure 1), establishes an average particle size of 9.59 nm.

**Figure 2:**
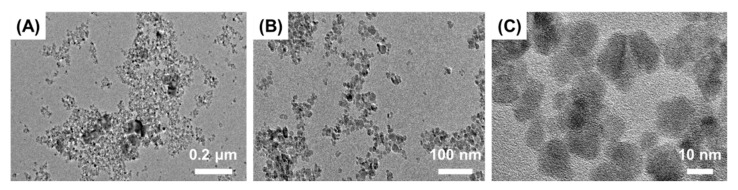
TEM images of Fe_3_O_4_ according to measurement magnifications (**A**) 15 k (**B**) 40 k (**C**) 200 K.

The morphology of the Fe_3_O_4_ particles was further identified by TEM, images for which are presented in Figure 2. Most of the Fe_3_O_4_ nanoparticles were quasi-spherical in shape with a diameter of 10 nm or less and formed aggregates that ranged in size from several hundreds of nm to several μm. The individual particle size obtained in the TEM analysis matched well with the sizes indicated by the XRD analyses. These results are similar to Kwon et al., who also reported on the properties of Fe_3_O_4_ [29]. The specific surface area of Fe_3_O_4_ obtained by BET analysis was 93.82 m^2^/g. 

### 3.2. The Catalytic Role of Fe_3_O_4_ in TC Degradation

**Figure 3:**
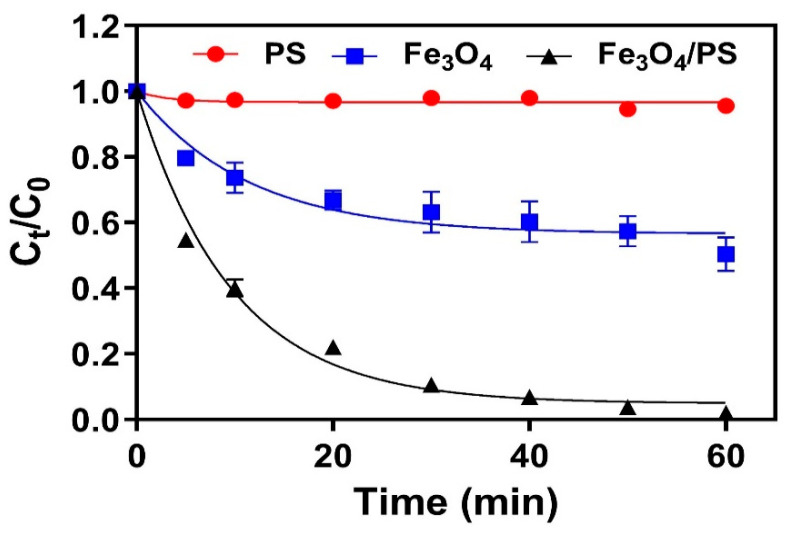
The normalised concentration of TC removed by PS, Fe_3_O_4_, and Fe_3_O_4_/PS over experimental time ([Fe_3_O_4_] = 1.0 g/L, [PS] =1.0 mM, [TC] = 41.6 μM, pH = 4.8).

The catalytic activity of Fe_3_O_4_ when activating PS and subsequently degrading TC was evaluated by monitoring TC removal in the presence or absence of PS and Fe_3_O_4_. As shown in Figure 3, PS alone resulted in only 4.4% TC removal efficiency after a one-hour reaction, while a comparably higher removal efficiency of 49.7% was observed when the only Fe_3_O_4_ was used. The dominant removal of TC by Fe_3_O_4_ was likely due to adsorption by the Fe_3_O_4_ surface, which is in line with the results from some previous similar studies [16,26]. This was further confirmed by the HPLC chromatography results for samples in the batch experiment with Fe_3_O_4_ alone, which did not show any intermediate peaks (Figure S1).

In comparison to the above results, reactions conducted with both Fe_3_O_4_ and PS exhibited much higher TC removal efficiencies of 90.0% in 30 min and 98.1% in 1 h. An HPLC chromatogram of the reaction solution revealed several intermediate peaks, thus establishing that oxidation is involved in TC removal (Figure S2). This result indicated that the enhanced removal efficiency in Fe_3_O_4_/PS was due to the oxidative degradation of TC. The concentration of total organic carbon (TOC) in the reaction solution also decreased by more than 50%, indicating that TC species were oxidatively decomposed by catalytic reactions. The degradation kinetic was further interpreted by the pseudo first-order kinetic. The high linearity (R^2^ = 0.9875) of the fitted line indicates that the reaction had pseudo first-order kinetics, which has been widely observed for similar catalytic systems [26]. The k_obs_ for the Fe_3_O_4_/PS system was calculated as 0.1038 min^−1^. Soluble iron concentration was maintained at less than 0.3 mg/L throughout the experiment for all samples, thereby verifying the high stability of Fe_3_O_4_ during the catalytic reaction.

### 3.3. The Effects of Reaction Conditions on TC Removal in Fe_3_O_4_/PS 

#### 3.3.1. Effect of Fe_3_O_4_ Concentration

**Figure 4:**
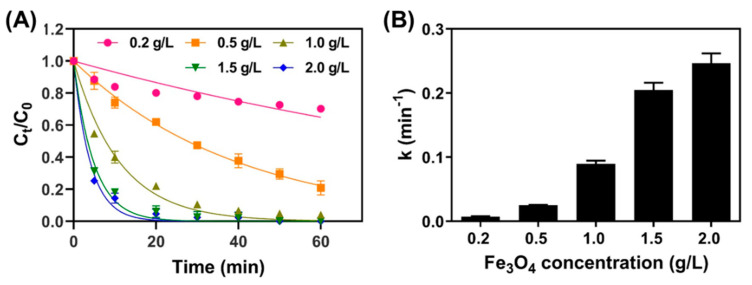
(**A**) TC removal plotted over time for systems with various Fe_3_O_4_ concentrations ([PS] = 1.0 mM; [TC] = 41.6 μM; pH = 4.8), and (**B**) the pseudo first-order rate constants of TC were obtained from the fitting the results in Figure 4A.

As discussed in Section 3.2, Fe_3_O_4_ was proven as an efficient catalyst for activating PS in order to remove TC. The effect of catalyst dosage on TC removal by Fe_3_O_4_/PS with various initial Fe_3_O_4_ concentrations (0.2–2.0 g/L) was studied. Initial concentrations of TC and PS were 41.6 μM and 1 mM, respectively. As presented in Figure 4A, the removal of TC increased when increasing the initial Fe_3_O_4_ concentration. For example, when 0.2 g/L, 0.5 g/L, and 1.0 g/L of Fe_3_O_4_ were used, the removal efficiencies of TC at a 60-min reaction time were 29.8%, 79.2%, 95.9%, respectively, and full removal of TC within our detection limits was achieved at Fe_3_O_4_ concentrations of 1.5 g/L or 2.0 g/L. In Figure 4B, the pseudo first-order rate constants gradually increased from 0.0039 to 0.1309 min^−1^ along with an increase in initial Fe_3_O_4_ concentration. The enhanced kinetic constants in increased Fe_3_O_4_ addition were attributed to the adsorption and oxidation. The higher catalyst concentrations accelerate TC decomposition, which can be explained by the greater concentration of active sites at higher Fe_3_O_4_ concentrations. Moreover, the large numbers of active sites also enhance the adsorption of TC and PS molecules to Fe_3_O_4_, leading to higher reaction kinetics [32]. 

#### 3.3.2. Effect of PS Concentration

**Figure 5:**
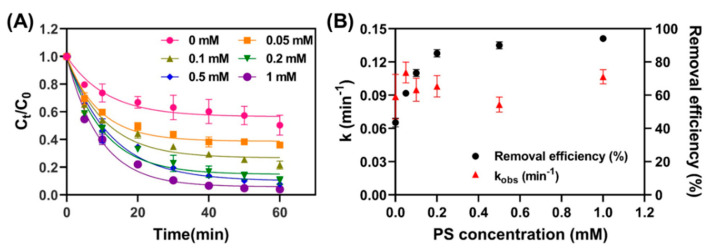
(**A**) Plotted TC removal efficiency versus PS concentration ([Fe_3_O_4_] = 1.0 g/L; [PS] = 1.0 mM; [TC] = 41.6 μM; pH = 4.8), and (**B**) plotted reaction rate coefficient against PS concentrations.

Similar to the batch study of the effect of Fe_3_O_4_ concentration on TC removal in the above section, the impact of the initial concentration of PS (0 to 1 mM) on TC removal in Fe_3_O_4_/PS was also investigated. As presented in Figure 5A, a system without the addition of PS had a TC removal efficiency of 43.5%, but this increased to 63.9% even with a relatively low 0.05 mM PS concentration. Further increasing the PS concentration to 1 mM PS dramatically raised TC decomposition efficiency to 95.9%. This distinct improvement in efficiency at higher PS concentrations possibly originates from the greater reactive radical generation apparent at higher PS concentrations [28,33].

Based on the kinetic fitting results in Figure 5A, the corresponding rate constants and removal efficiencies of TC under different PS initial concentrations were obtained (Figure 5B). PS concentration did not significantly affect the rate constant, in comparison to the impactor degree of Fe_3_O_4_ detailed in Section 3.3.1. Interestingly, TC removal efficiency after 60 min of reaction increased from 43.5 to 85.2% when PS concentration increased from 0 to 0.2 mM; thereafter, removal efficiency remained stable at above 0.2 mM. 

These results clearly indicate that the catalyst concentration determines the reaction rate in the heterogeneous Fenton system. By comparison, PS does not significantly influence the reaction rate, because PS is an oxidizing agent that is decomposed by the catalyst. In addition, excess PS hinders the overall reaction, due to the generation of sulfate anions via scavenging reactions among radicals, as shown in Equations (6) and (7) [12,34].

SO_4_^•−^ + SO_4_^•−^ → S_2_O_8_^2^^−^ (6)

SO_4_^•−^ + S_2_O_8_^2^^−^ → SO_4_^2−^ + S_2_O_8_^•−^ (7)

#### 3.3.3. Effect of Initial pH

**Figure 6:**
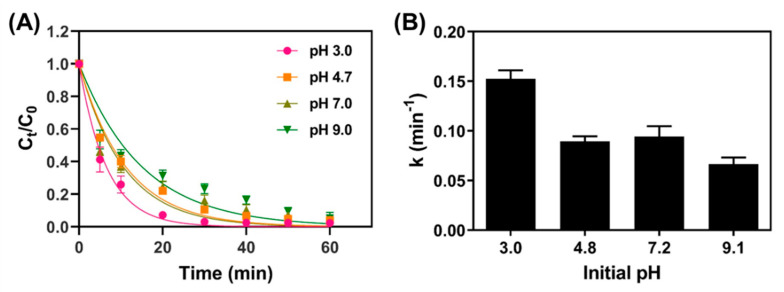
(**A**) TC removal efficiency for samples with different initial pH conditions ([Fe_3_O_4_] = 1.0 g/L; [PS] =1.0 mM; [TC] = 41.6 μM), and (**B**) reaction rate coefficient versus pH.

The heterogeneous Fenton reaction is influenced by the solution’s pH, because the dissolution of ferrous iron (Fe^2+^) depends on pH. Batch experiments under different pH conditions, ranging from 3.0 to 9.0, were conducted to understand their impact on the removal efficiency of TC by Fe_3_O_4_/PS. As presented in Figure 6, TC removal increased under low pH conditions, and the highest rate constant (0.1530 min^−1^) was obtained at pH 3.0. The rate constant decreased under alkaline conditions, and the lowest rate coefficient value of 0.066 min^−1^ was observed at pH 9.0. Previous reports demonstrated that when pH > 4.0, the concentration of soluble Fe^2+^ ions decreased, due to the formation of Fe^2+^ complexes and precipitation; subsequently, the activation rates for PS and SO_4_^•^^−^ production decreased [26]. In alkaline pH conditions, SO_4_^•−^ radicals converted into •OH radicals, thus serving as scavengers for SO_4_^•−^ via the reactions shown in Equations (8) and (9) below [35,36]:

SO_4_^•^^−^ + •OH → HSO_4_^−^ + 0.5O_2_ (8)

SO_4_^•^^−^ + H_2_O → SO_4_^2^^−^ + •OH + H^+^ (9)

However, the change in the reaction rate was not as dramatic as seen in similar studies [34,37], because, in our study, pH was only adjusted accordingly before starting the batch experiments, unlike the previous literature that used a pH buffer throughout the experiments. As a result, the initial pH values of 3.0, 4.8, 7.2, and 9.1 applied to the batch experiment were changed to final pH values of 3.0, 4.1, 4.2, and 4.5, respectively. No drop in pH was observed for samples with an initially low pH, while samples with higher initial pH values experienced more significant drops in this regard. These decreases in pH can be explained by the production of carboxylic acid products and the decomposition of sulfate salts [38,39]. 

### 3.4. Effect of the Water Matrix

As mentioned in the introduction, the water matrix effect of actual wastewater on TC removal by Fe_3_O_4_/PS system have not been reported; therefore, we examined the effect of the water matrix on the removal of TC by Fe_3_O_4_/PS by using municipal effluent wastewater from a local WWTP, while ultrapure water was used in the comparison batch study (Figure S3). 

The rate constants were similar in both batch studies, namely 0.100 min^−1^ in the municipal effluent and 0.107 min^−1^ in the ultrapure water, but removal efficiency at 60 min was significantly different at 88.3% and 96.0%, respectively. This clearly demonstrates a key role of the water matrix on the heterogeneous Fenton reaction for TC degradation. The consumption of PS in the municipal effluent was comparatively greater, due to the presence of other organic and inorganic matter, which by comparison did not affect the reaction rate. Additional experiments were conducted to interrogate how Fe_3_O_4_ and PS concentration influence TC removal in municipal effluent. The results of these experiments are summarized in Table 2. 

Overall, TC removal efficiency was lower in the municipal effluent than that in the ultrapure water, thereby suggesting that the generated oxidative radicals were consumed by organic/inorganic matter in the effluent and TC, which were competing with each other. Interestingly, when only the PS was introduced without Fe_3_O_4_, relatively higher TC removal efficiency was observed in the batch experiments treating the municipal effluent (42.5%) than those treating ultrapure water (4.4%). This may have been the result of the presence of metal cations (Mn, Cu, Ni) in the municipal effluent (Table S1) acting as persulfate activators (Equation (10)) [40,41,42]. Li et al. (2016) reported that the very low concentration of Mn^2+^, such as 0.5 mg/L, was enough to produce the hydroxyl radicals in the presence of hydrogen peroxide [43]. Moreover, organic matters, such as quinones and phenols, can also activate PS for subsequent degradation of organic matters, as reported by Fang et al. (2013) and Ahmad et al. (2013) [44,45].

S_2_O_8_^2^^−^ + M^n+^ → M^n+1^ + SO_4_^2−^ + SO_4_^•−^ (10)

When the initial PS concentration was fixed at 1 mM, increasing Fe_3_O_4_ concentrations also led to improvements in TC removal efficiencies, namely 42.5%, 54.2%, and 78.3% at 0 g/L, 0.5 g/L, and 1.0 g/L of Fe_3_O_4_, respectively. A similar trend was observed in ultrapure water. The effect of PS concentration was also probed in municipal effluent. In ultrapure water, increasing the dosage of PS from 0 to 0.2 mM yielded a logarithmic increase in TC degradation efficiency, which plateaued at PS concentrations from 0.2 to 1.0 mM. By comparison, in municipal effluent, TC removal efficiency gradually increased with PS content at low PS concentrations (0 to 0.5 mM), albeit it sharply increased at 1.0 mM. The low enhancement of TC removal efficiency with greater PS concentration is likely the result of TC competing with other organic and inorganic matter in the municipal effluent, which consumes radical species. Sulfate radicals can be scavenged at high-pH, organic carbons, and various anionic species, including Cl^−^, HCO_3_^−^, and CO_3_^2^^−^ that are present in effluent via the reactions in Equation (11) to (13) [40,46]. TOC removal efficiencies were 50 to 17% for ultrapure water and municipal effluent, respectively, indicating that TC degradation was hindered by organic and inorganic matter in the municipal effluent. The consumption of oxidative radicals by such compounds in the municipal effluent resulted in lower TC removal efficiencies:

SO_4_^•−^ + Cl^−^ → Cl^•^ + SO_4_^2−^ (11)

SO_4_^•−^ + HCO_3_^−^ → HCO_3_^•^ + SO_4_^2−^ (12)

SO_4_^•−^ + HCO_3_^−^ → CO_3_^•−^ + SO_4_^2−^ (13)

**Figure 7:**
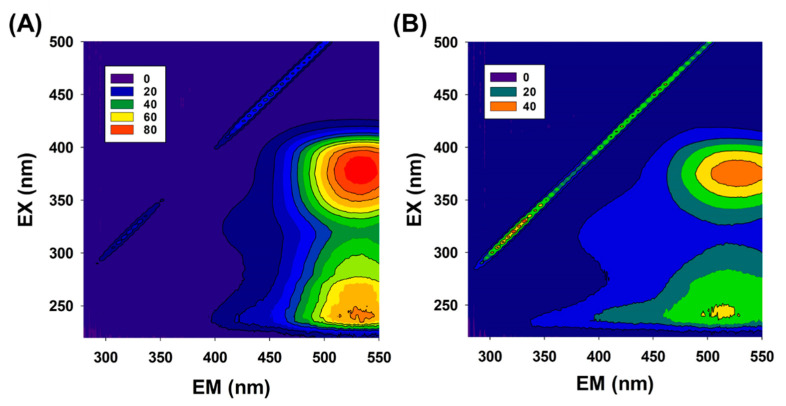
Excitation-emission matrix results acquired from the municipal effluent spiked with TC, before (**A**) and after (**B**) treating with Fe_3_O_4_/PS [Fe_3_O_4_] = 1 g/L, [PS]_0_ = 1 mM, [TC]_0_ = 41.6 μM).

Changes in organic matter with Fe_3_O_4_/PS were further analyzed by using excitation-emission matrix (EEM) fluorescence analyses. EEM data collected from TC-spiked municipal effluent (Figure 7A) show peaks in the 360–380 nm/520–540 nm (excitation/emission) and 230–240 nm/520–540 nm (excitation/emission) regions, corresponding to humic acid-like and fulvic acid-like groups, respectively. After 1 h of the TC degradation reaction (Figure 7B), overall peak intensity decreased by approximately half, indicating the degradation of organic matter during the Fe_3_O_4_/PS experiments. Moreover, peak intensities in the 230–240 nm/340–400 nm region comparatively increased after the reaction, thus indicating the presence of low-molecular-weight organic substances such as tryptophan-like and other protein-like compounds [47,48]. 

The EEM matrices were further analyzed by the humification index (HIX), which is the ratio of the integral of the emission spectrum (excited at 255 nm) over the spectral range of 434 to 480 nm to the integral of the emission spectrum over the spectral range of 330 to 346 nm (also excited at 255 nm) [49]. High-molecular-weight organic compounds are characterized by high HIX values. Table 3 summarizes the HIX values obtained for several water samples, municipal effluent, municipal effluent spiked with TC before the Fe_3_O_4_/PS experiment, and Fe_3_O_4_/PS-treated water. The HIX value was 6.51 for the municipal effluent, and this dramatically increased to 21.53 upon adding TC (41.6 μM). The HIX value for the Fe_3_O_4_/PS-treated water was relatively lower at 7.55, which is similar to the HIX value for municipal effluent. 

Similar trends were observed in the SUVA_254_ value, which is the ratio of decadal absorbance at 254 nm to the dissolved organic carbon concentration (DOC). Typically, the SUVA_254_ ranges from 1.2 to 2.6 L/mg·m for secondary effluents [50]. The SUVA_254_ value for the municipal effluent in this study was 1.51 L/mg·m, which falls within the reported range. By comparison, municipal effluent spiked with TC exhibited a relatively greater SUVA_254_ of 4.13, which subsequently reduced to 2.20 after Fe_3_O_4_/PS treatment. As the HIX and SUVA_254_ are measures of the aromaticity of organic matter, we conclude that the aromatic rings in the TC molecule degraded during the reaction promoted by the Fe_3_O_4_/PS. The reduced aromaticity resulting from the Fe_3_O_4_/PS agrees well with related studies reporting on the mechanism of TC degradation in the Fenton reaction [21,51]_._

### 3.5. Transformation Products and Proposed Pathways 

**Figure 8:**
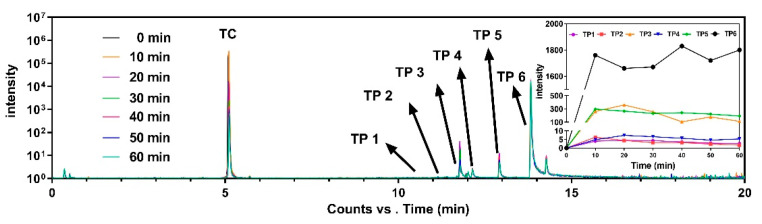
The overlapped total ion chromatogram for the experimental time for the intensity development of TC and its transformation products eluted at different retention times. The detailed intensity development for six transformation products during the experiments is top right.

Intensity development during the reaction between TC and Fe_3_O_4_/PS for the TC peak eluted at 5.09 min, and six major peaks inferred to the transformation products (TPs) can be seen in total ion chromatography in Figure 8 and Figure S4. The TPs were found at elution times ranging from 10 to 14 min, namely, TP 1(*m*/*z* = 344, 10.69 min), TP 2 (*m*/*z* = 358, 11.06 min), TP 3 (*m*/*z* = 274, 11.77 min), TP 4 (*m*/*z* = 290, 12.02 min), TP 5 (*m*/*z* = 256, 12.92 min), and TP 6 (*m*/*z* = 284, 13.82 min). TC peak intensity significantly decreased over time, while the TP 6 peak intensity increased over time. Unlike TP 6, the intensities of TP 1–5 peaks initially increased but then decreased. These TPs with their predicated molecular structures, and the corresponding mass spectrum intensities, are illustrated in Table S2 and Figure S5. 

**Figure 9:**
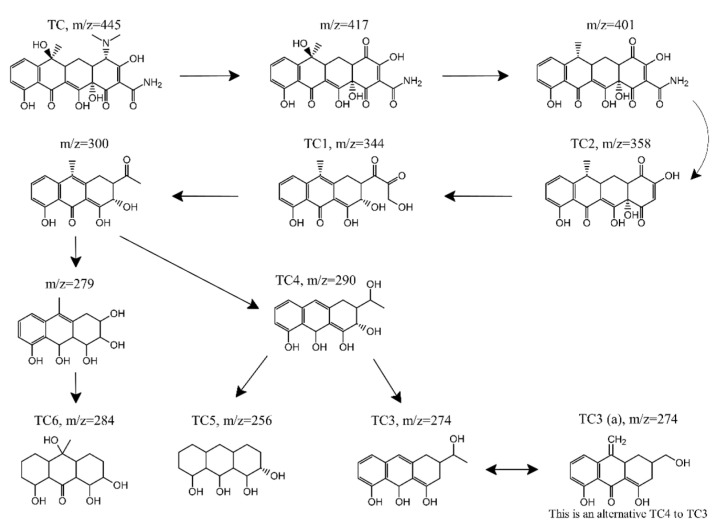
A possible TC degradation pathway in the Fe_3_O_4_/PS system.

A possible TC degradation pathway is proposed in Figure 9. First, the formation of TP with an *m*/*z* = 417 was the result of losing dimethyl amino at C4 of TC (*m*/*z* = 445), due to low N-C bond energy [28]. TP with an *m*/*z* = 401 formed through the dehydration pathway from C6 of the TP with an *m*/*z* = 417 [52]. Through the deamidation reaction, namely, the loss of the acylamino group at C1 of TP with an *m*/*z* = 401 [53], the formation of TP 2 (*m*/*z* = 358) was observed. Thereafter, TP 1 (*m*/*z* = 344) was found, due to the cleavage of the carboatomic ring of TP 2 [54], followed by decarboxylation, which converted TP 1 to the TP with an *m*/*z* = 300 [55]. After dimethyl and deformaldehyde process by H addition, TP with an *m*/*z* = 279 was obtained, while the formation of TP 6 (*m*/*z* = 284) was attributed to TP with an *m*/*z* = 279 of decarboxylation and H addition reaction to C-H double bond on benzene ring as well as carboxyl addition. TP 6 was also identified as one of the transformation products for previous references using the photocatalyst to remove TC [56,57]. Unlike the pathway from an *m*/*z* = 300 to an *m*/*z* = 284, TP 4 (*m*/*z* = 290) was formed by adding dimethyl and H to the C-O double bond of the TP with an *m*/*z* = 300. Based on TP 4, TP 3 was formed due to its decarboxylation, while TP 5 was obtained through its dimethyl decarboxylation and H addition reaction to a C-H double bond on a benzene ring. When compared to TP 3, TP 3 (a) had the same *m*/*z* as TP 3 but a different molecular structure and was directly derived from TC. TP 3 (a) was also found in a previous study using a catalyst regenerated from Fenton sludge containing heavy metal, in order to active persulfate for TC degradation [53].

## 4. Conclusions

This study presents a new understanding of the degradation of TC via a heterogeneous Fenton system using Fe_3_O_4_ and persulfate. Fe_3_O_4_ was successfully synthesized by using the coprecipitation method under alkaline conditions, and the resulting materials were characterized via XRD, TEM, and BET analyses. The catalytic roles of Fe_3_O_4_ for persulfate activation and TC degradation were evaluated by monitoring TC reduction in the presence or absence of persulfate and Fe_3_O_4_. TC was removed from solutions by adsorption onto Fe_3_O_4_ surfaces, where the TC was then oxidatively degraded by the Fenton reaction. The effects of various reaction conditions, including Fe_3_O_4_ concentration, PS concentration, and the initial pH, were further interrogated to understand the reaction mechanism. The kinetics of TC decomposition gradually increased in line with an increase in Fe_3_O_4_ concentration, thus indicating the catalytic role of Fe_3_O_4_. Increasing the concentration of PS influenced TC removal efficiency but not TC removal kinetics, because persulfate is an oxidizing agent that is decomposed by the catalyst. Excess PS hindered the overall reaction by scavenging sulfate radicals. Lower initial pH conditions enhanced the overall reaction kinetic by increasing Fe^2+^ generation. The effect of the water matrix was further investigated by using WWTP effluent. Overall, TC removal efficiency was lower in the effluent versus ultrapure water, due to TC competing with organic and inorganic matter that consumes generated oxidative radicals. DOM analyses, e.g., EEM and SUVA_254_, revealed that reactions promoted by the HFS break the aromatic ring moieties of TC molecules. The oxidative degradation of TC was nonetheless maintained in the effluent, and TC removal efficiency in WWTP effluent could be increased by raising the concentration of PS. The tetracycline degradation pathway through the magnetite/persulfate system was identified by using a liquid chromatograph-tandem mass spectrometer. Thus, the heterogeneous Fenton process with Fe_3_O_4_ and PS appears to have significant potential for use in removing and controlling micropollutants in wastewater.

## Figures and Tables

**Table 1:** Comparison of experimental conditions used in this research with literature.

Catalyst Type	Catalyst Dosage (g/L)	PS Dosage (mM)	[TC]_0_ (mg/L)	LC-MS Study	Water Matrix Tested	Ref.
Fe_3_O_4_	0.2~2	0.05~1	18.5	O	Municipal effluent	This study
Fe_3_O_4_	0.2	2	50	X	X (humic acid)	[21]
Fe_3_O_4_	0.1~1.5	15~150 (H_2_O_2_)	25	O	X	[22]
Fe_3_O_4_/biochar	0.1~0.5	0.1~20	5~40	O	X (inorganic ions; Cl^−^, SO_4_^2−^, NO_3_^−^, HCO_3_^−^, H_2_PO_4_^−^)	[25]
Fe_3_O_4_/biochar	0.5~2	2~16	20	O	X (inorganic ions; Cl^−^, NO_3_^−^, HCO_3_)	[26]
AC@Fe_3_O_4_	0.05~0.4	10~50	10~50	X	X	[27]
Fe_3_O_4_-CS	0.3~0.7	5~40 (H_2_O_2_)	44~111	O	X	[28]

**Table 2:** TC removal efficiencies in ultrapure water and municipal effluent according to various reaction conditions.

Fe_3_O_4_ Dosage (g/L)	PS Dosage (mM)	TC Removal Efficiency at 60 min (%)	
		Ultrapure Water	Municipal Effluent
0	1.0	4.4 ± 1.0	42.5 ± 3.3
0.5	1.0	79.2 ± 3.2	54.2 ± 2.1
1.0	0.0	49.7 ± 5.1	55.2 ± 5.9
	0.1	78.4 ± 2.1	53.6 ± 3.2
	0.2	89.7 ± 2.0	60.0 ± 0.4
	0.5	92.3 ± 0.7	64.2 ± 0.2
	1.0	95.9 ± 0.1	78.3 ± 1.3

**Table 3:** Humidification index and SUVA_254_ for the various water samples.

	Municipal Effluent	Municipal Effluent Spiked with TC	Fe_3_O_4_/PS Treated
HIX	6.51	21.53	7.55
SUVA (L/mg·m)	1.51	4.13	2.20

## Data Availability

The data presented in this study are available on request from the corresponding author.

## Supplementary Materials

The following are available online at https://www.mdpi.com/article/10.3390/nano11092292/s1, Text S1: HPLC-MS/MS operational parameters for identifying the transformation product of tetracycline removed by magnetite/PS, Table S1: common parameters of municipal effluent wastewater, Table S2: transformation products (TPs) of tetracycline removed by Fe_3_O_4_/PS, Figure S1: HPLC peak when TC was removed by only Fe_3_O_4_, Figure S2: HPLC peak when TC was removed by Fe_3_O_4_/PS, Figure S3: reduction in TC concentration in (red) municipal effluent and (black) ultrapure water ([Fe_3_O_4_] = 1 g/L, [PS]_0_ = 1 mM, [TC]_0_ = 41.6 μM), Figure S4: the total ion chromatogram (HPLC-MS/MS) of tetracycline removed by Fe_3_O_4_/PS and its transformation products in samples taken over the reaction time, Figure S5: intensity of the fragment chart analysis relating to the transformation products of TC eluted at different retention times.
