# Supplementary material for: Oxidative Degradation of Tetracycline by Magnetite and Persulfate: Performance, Water Matrix Effect, and Reaction Mechanism
Source: Nanomaterials (Basel). 2021 Sep 3;11(9):2292. doi: 10.3390/nano11092292 (PMC8471070; doi:10.3390/nano11092292)
Supplement: Supplementary file 1 [file nanomaterials-11-02292-s001.zip › nanomaterials-1345386-supplementary.pdf]

**Supplementary Information**

# Oxidative degradation of tetracycline by magnetite and persulfate: performance, water matrix effect, and reaction mechanism

Deokhui Lee[a], Soyeon Kim[a], Kai Tang[b] Michael De Volder[c], Yuhoon Hwang[a,1]

[a]Department of Environmental Engineering, Seoul National University of Science and Technology, Seoul 01811, Republic of Korea

[b]Department of Environmental Engineering, Technical University of Denmark, Konges-Lyngby, Denmark

[c]Institute for Manufacturing, Department of Engineering, University of Cambridge, Cambridge CB3 0FS, United Kingdom

## Table of contents

**Text S1.** HPLC-MS/MS operational parameters for identifying the transformation product of tetracycline removed by magnetite/PS

**Table S1.** Common parameters of municipal effluent wastewater

**Table S2.** Transformation products (TPs) of tetracycline removed by $Fe_3O_4$/PS

**Figure S1.** HPLC peak when TC was removed by only $Fe_3O_4$

**Figure S2.** HPLC peak when TC was removed by $Fe_3O_4$/PS

**Figure S3.** Reduction in TC concentration in (red) municipal effluent and (black) ultrapure water ($[Fe_3O_4]$ = 1g/L, $[PS]_0$ = 1 mM, $[TC]_0$ = 41.6 μM)

**Figure S4.** The total ion chromatogram (HPLC-MS/MS) of tetracycline removed by $Fe_3O_4$/PS and its transformation products in samples taken over the reaction time

**Figure S5.** Intensity of the fragment chart analysis relating to the transformation products of TC eluted at different retention times

**Text S1. HPLC-MS/MS operational parameters for identifying the transformation product of tetracycline removed by $Fe_3O_4$/PS**

The HPLC system was a Vanquish Flex, provided by Thermo Fisher Scientific, USA, and the column used for chromatographic separation was a C18 column (4.6 x 50 mm, 3.5 µm, Eclipse Agilent, USA). The temperature of the HPLC column oven was 35ºC, and the pump flow rate was 0.3 mL/min. The sample injection volume was 10 µL, and the HPLC gradient was formed by changing the mix ratio of Milli-Q water, including 0.1% of formic acid (solvent A), and methanol, including 0.1% of formic acid (solvent B). The tandem mass spectrometer equipped with a triple quadrupole was a TSQ Quantis from Thermo Fisher Scientific, USA. For the full-scan mode, the gradient began with 5% B, followed by a linear increase of gradient B up to 100% within 15 mins, following which 100% B was kept running for another 5 mins. Initial gradient conditions were also re-established for 3 mins. A positive ESI ionization mode (ESI+) was applied, and the spray voltage was fixed to 4000 V. The scanning m/z range was from 60 Da to 600 Da

**Table S1.** Common parameters of municipal effluent wastewater.

| | Concentrations |
|---|---|
| pH | 6.89 |
| TOC (mg/L) | 12.25 |
| $Cl^-$ (mg/L) | 77.04 |
| $NO_3^-$ (mg/L) | 39.08 |
| $SO_4^{2-}$ (mg/L) | 50.88 |
| $Na^+$ (mg/L) | 52.55 |
| $K^+$ (mg/L) | 11.63 |
| $Mg^{2+}$ (mg/L) | 6.43 |
| $Ca^{2+}$ (mg/L) | 32.2 |
| $Mn^{2+}$ (μg/L) | 31.42 |
| $Co^{2+}$ (μg/L) | 0.17 |
| $Ni^{2+}$ (μg/L) | 8.01 |
| $Cu^{2+}$ (μg/L) | 0.83 |

**Table S2.** Transformation products (TPs) of tetracycline removed by $Fe_3O_4$/PS.

| Products | m/z value | Retention time (min) | Formula | Structure |
|---|---|---|---|---|
| **Tetracycline** | 445 | 5.09 | $C_{22}H_{24}N_2O_8$ | 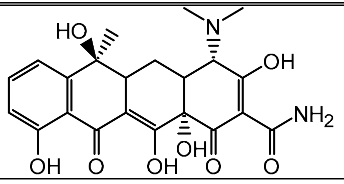 |
| **TP 1** | 344 | 10.69 | $C_{18}H_{16}O_7$ | 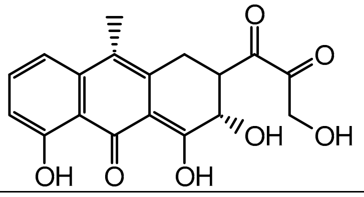 |
| **TP 2** | 358 | 11.06 | $C_{19}H_{16}O_7$ | 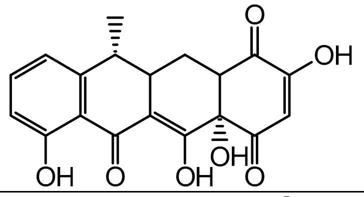 |
| **TP 3** | 274 | 11.77 | $C_{16}H_{17}O_4$ | 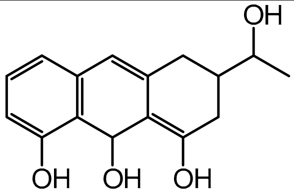 |
| | | | $C_{16}H_{15}O_4$ | 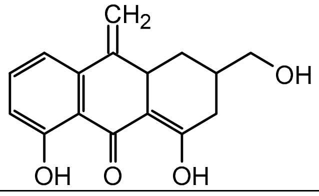 |
| **TP 4** | 290 | 12.02 | $C_{16}H_{18}O_5$ | 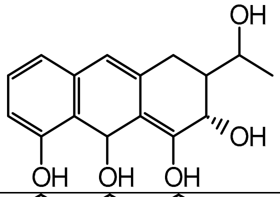 |
| **TP 5** | 256 | 12.92 | $C_{14}H_{24}O_4$ | 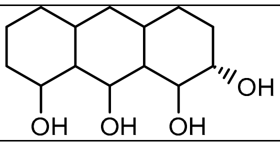 |
| **TP 6** | 284 | 13.82 | $C_{15}H_{24}O_5$ | 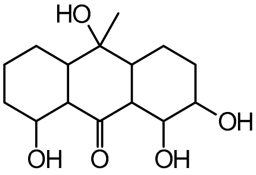 |

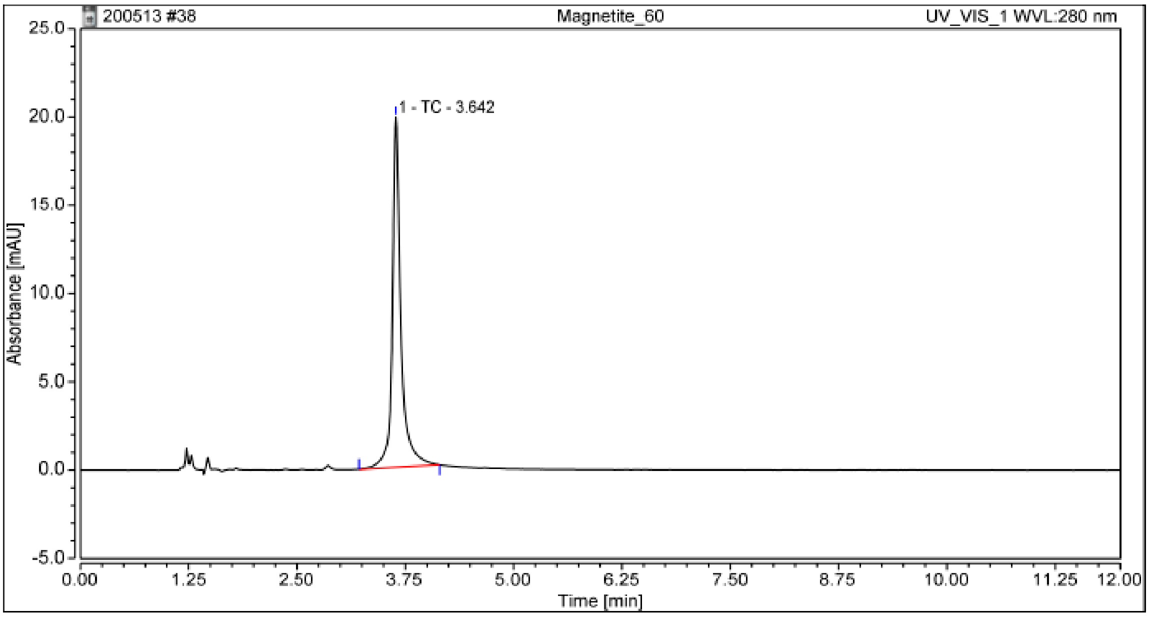


**Figure S1.** HPLC peak of TC treated by only $Fe_3O_4$.

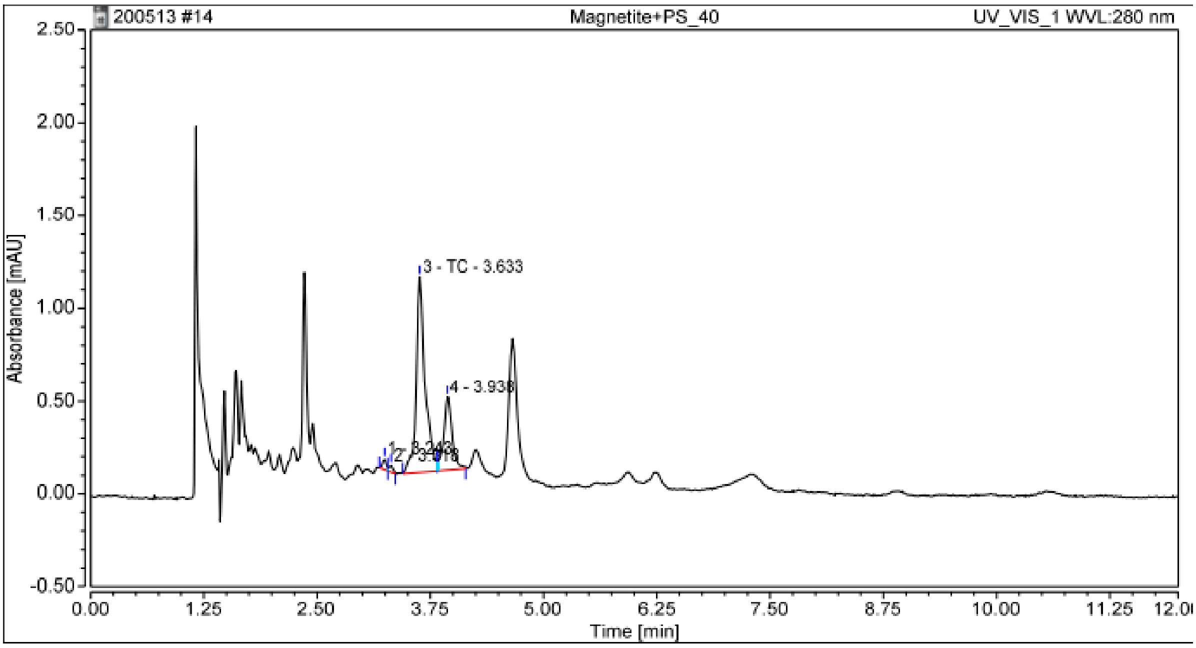


**Figure S2.** HPLC peak of TC treated by $Fe_3O_4$/PS.

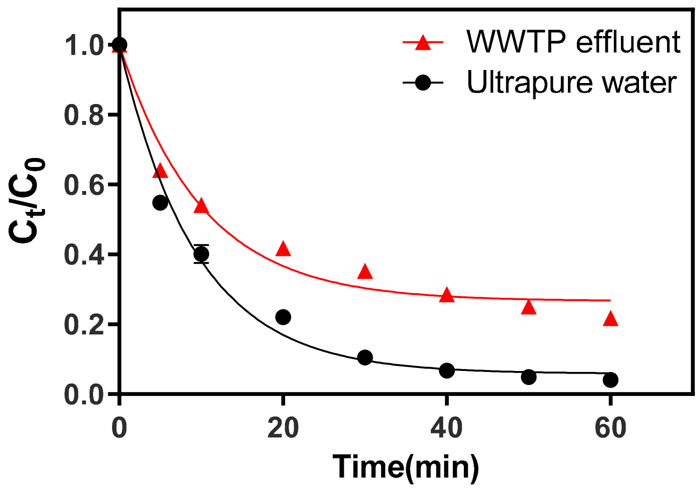


**Figure S3.** Reduction in TC concentration in (red) WWTP effluent and (black) ultrapure water ([$Fe_3O_4$] = 1g/L, $[PS]_0$ = 1 mM, $[TC]_0$ = 41.6 μM).

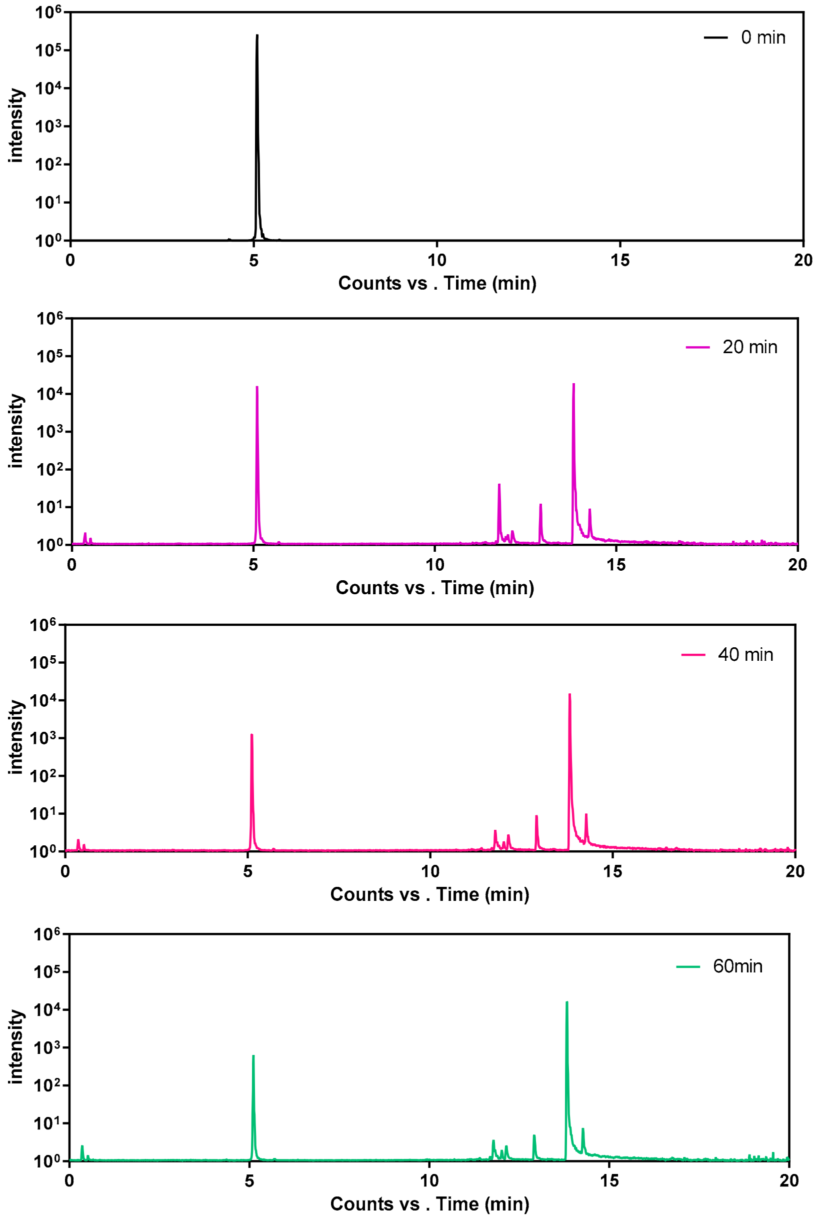


**Figure S4.** The total ion chromatogram (HPLC-MS/MS) of tetracycline removed by $Fe_3O_4$/PS and its transformation products in samples taken over the reaction time

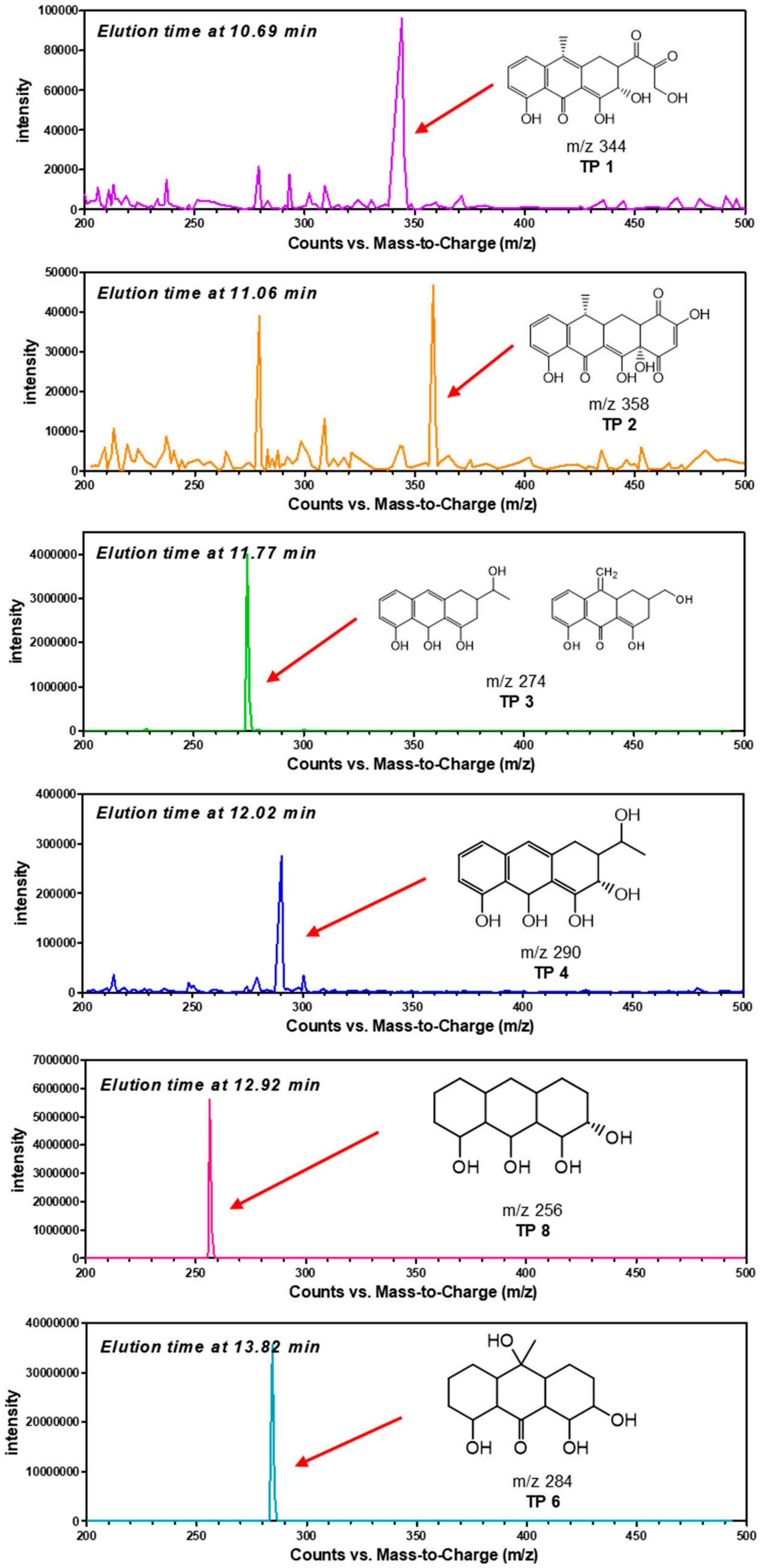


**Figure S5.** Intensity of the fragment chart analysis relating to the transformation products of TC eluted at different retention times.
